# Equatorial Pacific forcing of western Amazonian precipitation during Heinrich Stadial 1

**DOI:** 10.1038/srep35866

**Published:** 2016-10-25

**Authors:** Yancheng Zhang, Xu Zhang, Cristiano M. Chiessi, Stefan Mulitza, Xiao Zhang, Gerrit Lohmann, Matthias Prange, Hermann Behling, Matthias Zabel, Aline Govin, André O. Sawakuchi, Francisco W. Cruz, Gerold Wefer

**Affiliations:** 1 MARUM – Center for Marine Environmental Sciences, University of Bremen, Germany; 2 Alfred Wegener Institute Helmholtz Centre for Polar and Marine Research, Bremerhaven, Germany; 3 School of Arts, Sciences and Humanities, University of São Paulo, São Paulo, Brazil; 4 Department of Palynology and Climate Dynamics, Albrecht-von-Haller-Institute for Plant Sciences, University of Göttingen, Germany; 5 IPSL/LSCE, Laboratoire des Sciences du Climat et de l′Environnement (CEA-CNRS-UVSQ), Université Paris Saclay, Gif sur Yvette, France; 6 Institute of Geosciences, University of São Paulo, São Paulo, Brazil

## Abstract

Abundant hydroclimatic evidence from western Amazonia and the adjacent Andes documents wet conditions during Heinrich Stadial 1 (HS1, 18–15 ka), a cold period in the high latitudes of the North Atlantic. This precipitation anomaly was attributed to a strengthening of the South American summer monsoon due to a change in the Atlantic interhemispheric sea surface temperature (SST) gradient. However, the physical viability of this mechanism has never been rigorously tested. We address this issue by combining a thorough compilation of tropical South American paleorecords and a set of atmosphere model sensitivity experiments. Our results show that the Atlantic SST variations alone, although leading to dry conditions in northern South America and wet conditions in northeastern Brazil, cannot produce increased precipitation over western Amazonia and the adjacent Andes during HS1. Instead, an eastern equatorial Pacific SST increase (i.e., 0.5–1.5 °C), in response to the slowdown of the Atlantic Meridional Overturning Circulation during HS1, is crucial to generate the wet conditions in these regions. The mechanism works via anomalous low sea level pressure over the eastern equatorial Pacific, which promotes a regional easterly low-level wind anomaly and moisture recycling from central Amazonia towards the Andes.

Amazonia, host of the richest terrestrial biomes on Earth^1^,^2^,^3^, plays a fundamental role in the tropical water cycle^4^. Future possible changes of Amazonian precipitation that bear direct consequences on Amazon ecosystems^5^,^6^ and carbon storage^7^,^8^,^9^ are of great concern^10^. Analysis of observational data demonstrated a strong dependence of western Amazonian precipitation (e.g., the 2005 drought) on the Atlantic meridional sea surface temperature (SST) gradient^11^, but equatorial Pacific climate anomalies have also been related to Amazonian droughts and floods^12^,^13^. Potential decreases in the strength (by ca. 20–40%^14^) of the Atlantic Meridional Overturning Circulation (AMOC) under climate warming, which involve variations in both the Atlantic meridional SST gradient^15^ and the tropical eastern Pacific SST^16^, are rationally expected to affect Amazonian precipitation in the future. Past intervals when the AMOC underwent substantial reduction, such as Heinrich Stadial 1 (HS1, 18-15 ka before present, BP), provide valuable information on the response of South American precipitation to a weakened AMOC.

HS1 was characterized as the strongest AMOC perturbation over the last glacial period^17^ and significantly influenced tropical South American climate^18^,^19^,^20^,^21^. For example, a southward migration of the Intertropical Convergence Zone (ITCZ) during HS1, if compared to the Last Glacial Maximum (LGM, 23-19 ka BP), resulted in a considerable decrease of precipitation over northernmost South America^22^,^23^ and a substantial increase over northeastern (NE) Brazil^24^,^25^,^26^. To explain wet conditions in the Andes^27^,^28^,^29^ and southeastern (SE) Brazil^20^ during HS1, some authors proposed a strengthening of the South American summer monsoon (SASM) (Fig. 1a). Various freshwater-hosing experiments with climate models of different complexity levels (under both LGM^30^ and modern^31^ boundary conditions) successfully simulated the Atlantic ITCZ shift, but exhibited a large spread of rainfall patterns across western Amazonia. In addition, a growing number of studies also suggested a correlation between increased precipitation along the Andes and the El Niño Southern Oscillation (ENSO) during HS1^32^,^33^,^34^.

In this study, we integrate (i) a quality-flagged compilation of 107 published hydroclimate records from tropical South America and (ii) a suite of sensitivity experiments in an Atmospheric General Circulation Model (AGCM) to evaluate the impacts of HS1 (relative to the LGM) SST anomalies on tropical South American precipitation (see Materials and Methods). Our results show that SST changes over the eastern equatorial Pacific rather than the Atlantic are responsible for the increased precipitation over western Amazonia and the adjacent Andes during HS1.

## Results

### Compilation of hydroclimate records

**Figure 1:**
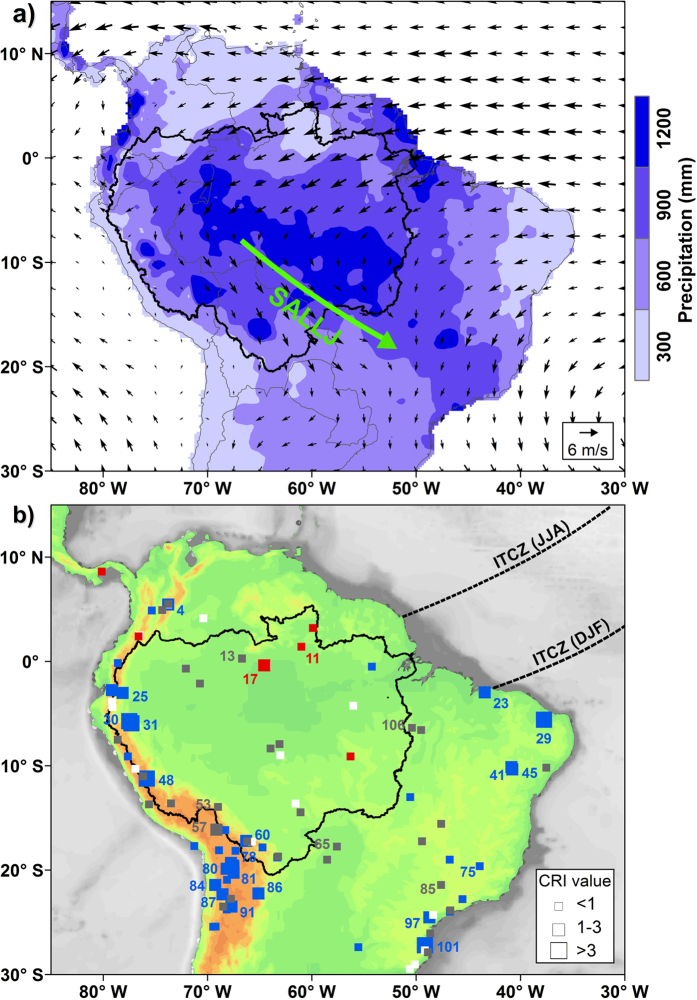
Precipitation and low level atmospheric circulation (**a**) and paleorecords compilation for tropical South America (**b**). (**a**) Long-term (1981–2010) averaged terrestrial precipitation (color scale) from the University of Delaware (http://climate.geog.udel.edu/~climate/) and 850 hPa wind field (vectors) from the NOAA/OAR/ESRL PSD (http://www.esrl.noaa.gov/psd/) during austral summer (December-January-February, DJF). Thick green arrow marks the South American low level jet (SALLJ). (**b**) Compilation of hydroclimate records, expressed as the difference between Heinrich Stadial 1 (HS1, 18-15 ka) and the Last Glacial Maximum (LGM, 23-19 ka). Symbol color indicates drier (red), wetter (blue), similar (grey) and unclear (white) conditions during HS1 in comparison to the LGM. Symbol size denotes the quality of the age model based on the chronological reliability index (CRI) (see Supplementary Information). Paleoclimate records with CRI values > 1 are numbered (Supplementary Table S1). Black dashed lines mark the schematic location of the Intertropical Convergence Zone (ITCZ) during austral summer (DJF) and austral winter (June-July-August, JJA). The Amazon River drainage basin is outlined by the black solid line in both panels (**a**,**b**). The map was plotted by using the ArcGIS software (version 10, https://software.zfn.uni-bremen.de/software/arcgis/).

Our compilation of paleomoisture difference between HS1 and the LGM indicates dry conditions to the north of the equator, but widespread wet conditions over the Andes, western Amazonia, NE and SE Brazil (Fig. 1b). Enhanced precipitation (or moisture) extends from the Ecuadorian Andes (e.g., Santiago Cave at ca. 3°S^27^) to the northern Chilean Andes (e.g., central Atacama Desert at 22°S–24°S^35^). The few available records from central Amazonia, characterized by low values of the chronological reliability index (CRI), exhibited in general dry climate during HS1 (Fig. 1b) (see Supplementary Information).

### Atmosphere model sensitivity experiments

**Figure 2:**
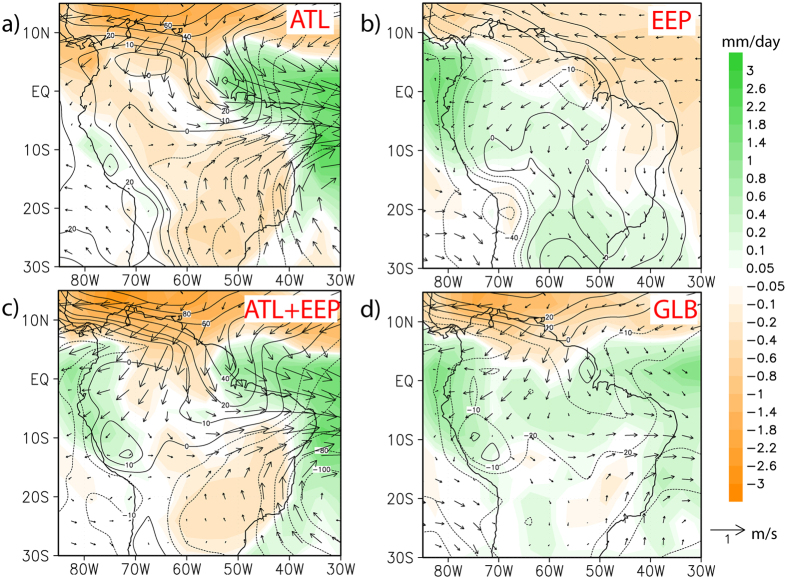
Results of the atmospheric model sensitivity experiments. Differences of simulated (ECHAM5) annual mean climate variables between Heinrich Stadial 1 and the Last Glacial Maximum for the (**a**) Atlantic (ATL) SST experiment, (**b**) eastern equatorial Pacific (EEP) SST experiment, (**c**) combined ATL + EEP experiment and (**d**) global (GLB) SST experiment (see Supplementary Information). Climate variables include rainfall (shaded, mm/day), 850 hPa wind field (vectors, m/s) and sea level pressure (contours, Pa). This map was plotted by using Grid Analysis and Display System (GrADS, Version 2.0.2, http://cola.gmu.edu/grads/grads.php).

The sensitivity experiments in this study were performed by using an atmosphere general circulation model (AGCM), the ECHAM5 (see Materials and Methods for a detailed design of model simulations). Driving the AGCM with global HS1 SST anomalies (see Supplementary Fig. S4) in the global SST experiment (Fig. 2d) shows comparable rainfall regimes to the ones simulated by the fully coupled atmosphere-ocean model^36^ (Supplementary Fig. S3). The ATL SST experiment that was forced by only Atlantic HS1 SST anomalies simulates a southward migration of the ITCZ, as evidenced by decreased rainfall over northernmost South America and increased rainfall over NE Brazil (Fig. 2a), but apparently fails to generate the wet conditions over western Amazonia. The EEP SST experiment (by applying only eastern equatorial Pacific HS1 SST anomalies) produces enhanced rainfall over western Amazonia together with the intensification of the northeast trade winds over central Amazonia and the South American Low Level Jet (SALLJ) (Fig. 2b), while the ITCZ displays no evident shift. The ATL + EEP SST experiment, in which we superimposed the eastern equatorial Pacific SST anomalies upon the Atlantic interhemispheric SST gradient, exhibits increased rainfall and easterly wind anomalies over western Amazonia (Fig. 2c), although dry conditions over SE Brazil are in contradiction to the GLB SST and the EEP SST experiments (Fig. 2b,d) as well as to our hydroclimate compilation (Fig. 1b).

## Discussion

During HS1, a stronger SASM associated with a change in the Atlantic interhemispheric SST gradient was commonly assumed to have triggered increased precipitation over the Amazonian Andes^27^,^28^,^29^. By contrast, our ATL SST experiment shows that the change in Atlantic interhemispheric SST gradient actually weakens the northeast trade winds over central Amazonia and the SALLJ (Fig. 2a), such that less moisture is transported from the tropical Atlantic towards western Amazonia and the adjacent Andes (Fig. 2a). Decreased precipitation over these areas as reproduced by the ATL SST experiment (Fig. 2a), however, conflicts with the prevailing wet conditions derived from our compilation (Fig. 1b). Thus, the Atlantic interhemispheric SST gradient alone is insufficient to explain the wet conditions over western Amazonia and the adjacent Andes during HS1, and contributions from other oceanic regions (e.g., tropical Pacific) should be taken into account.

The EEP SST experiment demonstrates that positive climatological SST anomalies over the eastern equatorial Pacific (Supplementary Fig. S4) are able to cause increased precipitation over western Amazonia and the adjacent Andes during HS1, probably in relation to enhanced northeast trade winds over central Amazonia and the SALLJ (Fig. 2b). Intensified northeast trade winds over central Amazonia, importantly, are also clearly identified in the ATL + EEP SST experiment (Fig. 2c). Remarkably, the wind field pattern over the western tropical Atlantic from the ATL + EEP SST experiment rather resembles that of the ATL SST experiment than of the EEP SST experiment (Fig. 2a,c). This result implies that in the ATL + EEP SST experiment, western Amazonia and the adjacent Andes still experienced an increased rainfall, although less equatorial Atlantic moisture was transported towards the Andes. These features agree well with the overall characteristics of our compilation (Fig. 1b), in particular with the presence of dry conditions over central Amazonia during HS1.

If the Atlantic meridional SST gradient was not the only driver for increased rainfall over the Amazonian Andes^37^,^38^,^39^, other processes must be involved. We turn to the SST increases of around 0.5-1.5 °C in the eastern equatorial Pacific during HS1, with the exception of minor SST decreases over coastal regions^40^,^41^ (Supplementary Fig. S4 and Table S2). These SST variations tend to yield low-pressure anomalies over the eastern equatorial Pacific, which then deepens the zonal sea level pressure (SLP) gradient between the Atlantic and the Pacific and strengthens the easterly flow anomaly over western Amazonia and the adjacent Andes (Fig. 2b–d). Such easterly wind anomalies together with the northeast trade winds over central Amazonia subsequently promote moisture recycling from central Amazonia towards the Andes, enhancing the evaporation-condensation along its pathway^42^ (as sketched in Supplementary Fig. S5). In fact, this mechanism was previously suggested to account for the wet Andean conditions during the LGM^42^, with a particular consideration of the Andes topography (Supplementary Fig. S9). The extent to which enhanced moisture recycling contributed to the wet conditions over the Amazonian Andes remains elusive, but our interpretation coincides with abundant evidence across the central Andes that substantiated increased proportions of regional-sourced moisture over HS1 and the LGM^32^,^33^,^34^,^35^,^43^,^44^,^45^,^46^.

Seasonal-scale SST changes in the eastern equatorial Pacific (Supplementary Figs S6 and S7) were often assigned to ENSO activity^47^. Because reconstructions of the ENSO variability across HS1 and the LGM were so far not well established from both numerical simulations^48^,^49^ and proxy data^50^,^51^,^52^, it is difficult to quantify the ENSO impact on South American precipitation during HS1. For instance, rainfall over NE Brazil and SE Brazil, which are today typically in strong negative and positive relationship with El Niño events^53^, indeed experienced similar wet patterns during HS1 (Fig. 1b). Analyses of instrumental data also suggested that climatological conditions over the eastern equatorial Pacific (e.g., related to ENSO^52^) may be linked to Atlantic climate forcing^54^,^55^. Eastern equatorial Pacific SST variations, probably a response to the weakened AMOC during HS1, are, nevertheless, crucial for triggering wet conditions over western Amazonia and the adjacent Andes (Fig. 1b).

Our ATL and ATL + EEP SST experiments (Fig. 2a,c) are unable to produce increased SE Brazilian rainfall as seen in the paleodata during HS1 (Fig. 1b). Interestingly, the GLB SST experiment (Fig. 2d), although forced by global SST anomalies (Supplementary Fig. S4), still cannot capture the wet conditions over SE Brazil. The SALLJ is weakened in both the GLB SST and the ATL + EEP SST experiments (relative to the EEP SST experiment), and thus seems unlikely to transport equatorial Atlantic moisture via western Amazon towards SE Brazil. In a recent paper, Kageyama *et al*.^30^ compared eleven freshwater-hosing experiments (under the LGM conditions) with six different fully coupled climate models, none of which, notably, showed increased rainfall over SE Brazil. Therefore, additional investigations on paleoclimate records and model simulations are necessary to clarify this point.

## Conclusion

Comparing a compilation of hydroclimate records and atmosphere model sensitivity experiments provides a deeper understanding of the influence of glacial North Atlantic climate on South American precipitation during HS1. An anomalous Atlantic meridional SST gradient, due to AMOC slowdown, drove a southward ITCZ shift leading to decreased precipitation over northernmost South America and increased precipitation over NE Brazil. The concomitant variations in eastern equatorial Pacific SST produced a negative SLP anomaly over the eastern tropical Pacific, which then deepened the SLP gradient between the Atlantic and the Pacific. As a result, it strengthened the northeasterly winds over the central and western Amazonia, enhancing moisture recycling over western Amazonia and the adjacent Andes.

Our results highlight that future changes in the eastern equatorial Pacific SST, as the AMOC weakens, will be of vital importance to affect western Amazonian precipitation. Depending on the magnitude of the AMOC slowdown under different global warming scenarios^10^,^14^, consideration of both the eastern equatorial Pacific and Atlantic SST variations may allow more accurate insights into the possible changes of Amazonian precipitation in the future.

## Materials and Methods

Paleomoisture (precipitation) difference between HS1 and the LGM over tropical South America was determined by compiling 107 published terrestrial hydroclimate records between 30°S–10°N and 80°W-35°W, including 53 lacustrine sediment cores, 10 alluvial deposits, 9 speleothems, 9 moraine landforms, 9 fauna remains, 7 shoreline deposits, 5 paleosol sequences, 3 paleodune profiles as well as 2 ice cores. Chronologies and proxies of all these paleorecords were used directly from their original references. To evaluate the dating quality of the selected hydroclimate records, we applied a chronological reliability index (CRI)^56^ based on (i) age model properties and (ii) sampling resolution of each record (detailed description is given in Supplementary Information). Higher CRI values indicate more reliable hydroclimate records (Supplementary Fig. S1). By referring to interpretations of proxies in each record individually, we identified four types of paleomoisture (precipitation) anomalies between HS1 and the LGM as: drier, wetter, similar or unclear (Supplementary Fig. S2, Table S1 and Supplementary Information).

To evaluate different regional contributions of climatological SST changes to South American precipitation anomalies between the LGM and HS1 (Supplementary Figs S3–S5), an atmospheric general circulation model (AGCM), the ECHAM5 (L19/T31, i.e., 19 vertical levels and 3.75° × 3.75° horizontal resolution)^57^ was employed. Since freshwater perturbation was the major forcing of the AMOC slowdown during HS1^30^,^58^, we used the LGM boundary conditions (i.e., orbital parameters, topography land-sea mask, ice sheet and greenhouse gas concentrations) to operate the experiments in this study. The LGM and HS1 control runs in the AGCM were forced by climatology monthly mean SST and sea ice cover from experiment LGMW and hosing experiment LGMW-0.2 Sv of the fully coupled general circulation model COSMOS (see ref. 36 for further details), respectively. To investigate the individual contributions of SST changes over different basins to South American precipitation anomalies during HS1, we conducted another three sensitivity experiments in which regional SST fields from the experiment LGMW−0.2 Sv^36^ were imposed upon the LGMW SST background, such as the Atlantic basin (30°S–80°N) (ATL), the eastern equatorial Pacific (180°E–~70°W, 25°S–25°N) (EEP, Supplementary Fig. S8) and a combination of ATL and EEP (ATL + EEP). The atmosphere model was integrated for 50 years for each model experiment and the last 30 years were taken to calculate climatological fields.

## Additional Information

**How to cite this article**: Zhang, Y. *et al*. Equatorial Pacific forcing of western Amazonian precipitation during Heinrich Stadial 1. *Sci. Rep.* **6**, 35866; doi: 10.1038/srep35866 (2016).

## Supplementary Material

Supplementary Information

Supplementary Information
