# Supplementary material for: Equatorial Pacific forcing of western Amazonian precipitation during Heinrich Stadial 1
Source: Sci Rep. 2016 Oct 25;6:35866. doi: 10.1038/srep35866 (PMC5078807; doi:10.1038/srep35866)
Supplement: Supplementary Information [file srep35866-s1.pdf]

Supplementary information

Equatorial Pacific forcing of western Amazonian precipitation during Heinrich Stadial 1

Yancheng Zhang*, Xu Zhang, Cristiano M. Chiessi, Stefan Mulitza, Xiao Zhang, Gerrit Lohmann, Matthias Prange, Hermann Behling, Matthias Zabel, Aline Govin, André O. Sawakuchi, Francisco W. Cruz, Gerold Wefer

* Correspondence to: yzhang@marum.de

1. Compilation of the hydroclimate records

For the compilation of published paleoclimatic records across tropical South America, we included the following archives: 53 lacustrine sediment cores (geochemistry, palynology and mineralogy), 10 alluvial deposits (geomorphology, palynology), 9 moraine deposits (glacial landforms), 9 speleothems (oxygen stable isotopes), 9 fauna remains (fossil rodent middens, palynology), 7 shoreline deposits (mollusc shells), 5 paleosol sequences (palynology, geochemistry), 3 paleodune profiles (geomorphology, luminescence ages), as well as 2 ice cores (oxygen stable isotopes) (Supplementary Fig.S1). Chronologies (e.g., using ^{14}C, U-Th, luminescence dating methods, as provided in Supplementary Table S1) and interpretation of the hydroclimate records agree to the original reference (Supplementary Table S1).

Fig.S1. Categorization of 107 hydroclimate records based on their chronological reliability index (CRI) values (data and detailed information are given in Supplementary Table S1). The map was plotted using the Microsoft Excel 2010 (https://www.marum.de/en/Microsoft.html).

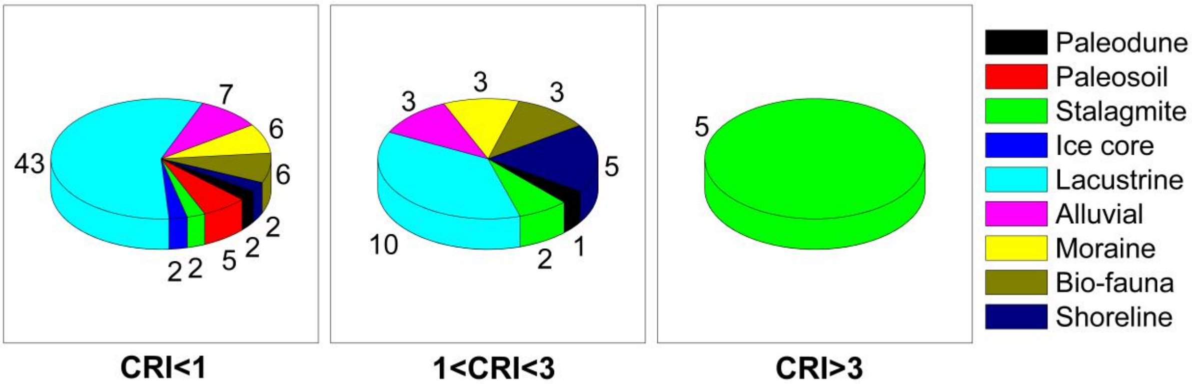

We used the time windows of 18-15 ka (ref. *S1*) and 23-19 ka (ref. *S2*) to define the Heinrich Stadial 1 (HS1) and the Last Glacial Maximum (LGM), respectively. For paleorecords with uncalibrated ^{14}C ages, the intervals of 15-12 ka ^{14}C ages and 20-16 ka ^{14}C ages were used to outline the HS1 and the LGM. Based on the chronological reliability index (CRI) (see below), we mainly used the paleorecords with CRI values over 1 to determine the overall pattern of the paleomoisture difference between HS1 and LGM. We acknowledge that the age controls based on uncalibrated ^{14}C ages may have large uncertainty, because the radiocarbon reservoir correction of terrestrial archives like sediment cores from lacustrine environment are less accurate due to local water-air CO_2 exchange (e.g., ref. *S3*). In the compilation, we included 28 paleoclimate records constrained by uncalibrated ^{14}C ages (with large age uncertainty as mentioned above), but note that only two of them are characterized by CRI values higher than 1 (e.g., sites No.85 and No.91 in Supplementary Table S1). Thus, the consideration of these paleorecords with uncalibrated ^{14}C ages does not modify our conclusions based on the compilation of South American hydroclimate records (Supplementary Fig.S2).

To calculate the CRI values, we employed the function established by Prado et al. (ref. *S4*) as:

$$\mathrm{CRI} = \frac{\mathrm{CA} + \mathrm{D} + \mathrm{R}}{3}$$

where CA (calibration) equals 1 (or 0) if age control points were (or not) calibrated; D (dating) is the number of age control points within HS1 and LGM, divided by 10; R (resolution) refers to the mean number of total samples per entire core length ratio, as given:

$$\mathrm{R} = \begin{cases} 0.1 \text{ for ratio between } 0.01 \text{ and } 0.1 \\ 0.2 \text{ for ratio between } 0.11 \text{ and } 0.2 \\ \quad\quad\quad\quad \cdots\ \cdots \\ 11.0 \text{ for ratio between } 10.01\text{and } 11.00 \end{cases}$$

Among the selected paleoclimate records, most speleothem records have high CRI values (≥3), while paleoclimatic archives from lacustrine environments (e.g., peat bog, peatland, swamp) or alluvial and moraine deposits generally show low CRI values (Supplementary Table S1, Fig.S1). Based on the interpretation of each paleorecord (see Supplementary Table S1 for further details), we identify the paleomoisture (precipitation) anomalies between HS1 and

LGM with three categories: 'drier', 'wetter' and 'similar'. For the paleorecords whose references did not allow a clear comparison (e.g., no information available on paleohydrology variations, or/and no age points available within HS1 or the LGM), we distinguish them as 'unclear' (Supplementary Fig.S2, detailed description is given in Supplementary Table S1).

Fig.S2. Types of South American hydroclimatic archives used to determine the difference between Heinrich Stadial 1 (HS1) and the Last Glacial Maximum (LGM) (data are provided in Supplementary Table S1). Red (blue) symbols denote a drier (wetter) HS1 than the LGM, while grey (white) symbols represent similar (unclear) conditions. Symbol sizes relate to the quality level established by chronological reliability index (CRI) (Supplementary Fig.S1). Numbers mark those records with CRI values > 1 (Supplementary Table S1) that constitute the base for the determination of paleomoisture difference between HS1 and the LGM. The map was plotted by using the ArcGIS software (version 10, https://software.zfn.uni-bremen.de/software/arcgis/).

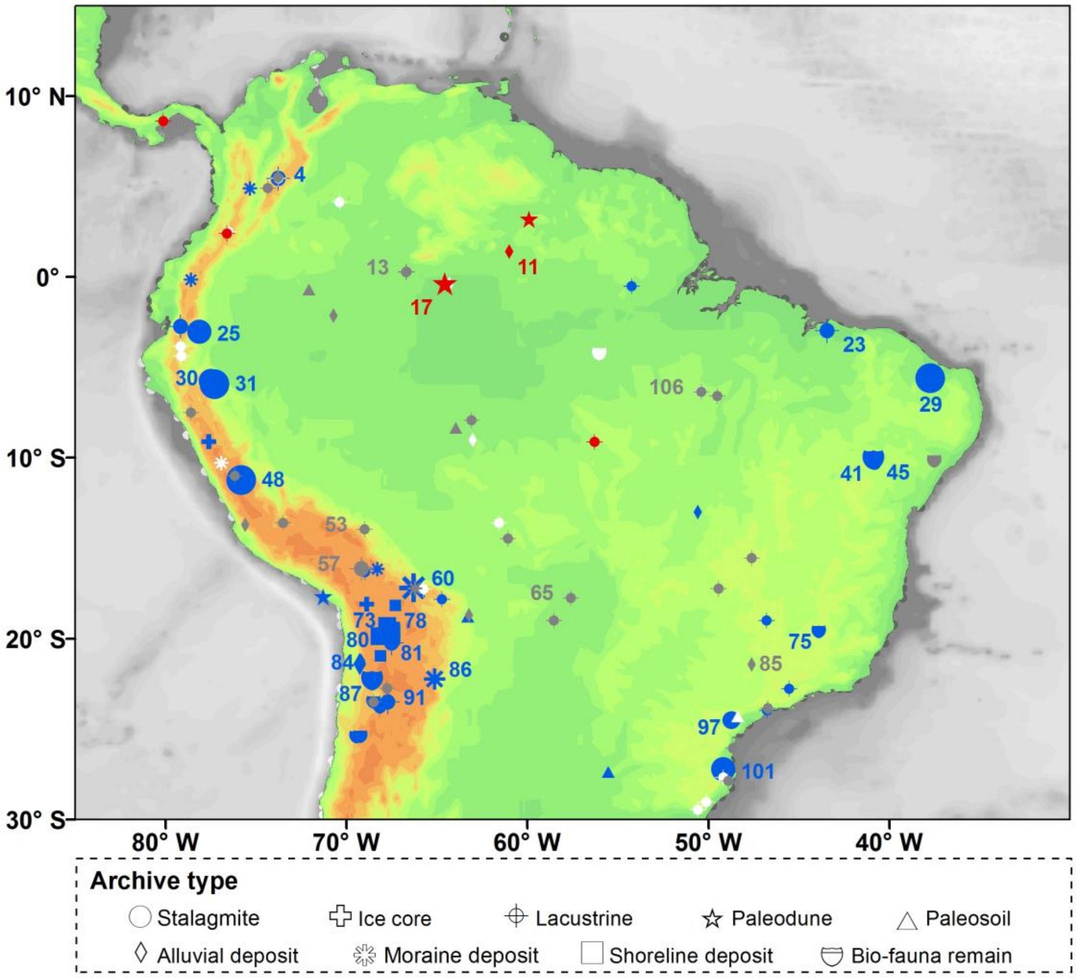

2. General dry HS1 vs. LGM conditions over central Amazonia

Based on palynological analyses, previous studies showed that the dry climate at Lagoa Verde (Hill of six lakes, site No.13; *S5-S6*) and Lagoa da Cachoeira (site No.106; *S7-S8*) was comparable between the LGM and HS1. The paleodune profile at Temedauí, Rio Negro (site No.17; *S9*) apparently demonstrated a slightly enhanced eolian activity during 17.2-16.4 ka compared to the period 22.8-22 ka, both periods being in intensified state relative to the Holocene. Similarly, the megafan at Viruá National Park (site No. 11, *S10*) suggested a high water discharge in wet seasons during 24-20 ka, while an extensive dry season was associated with C4 grassland development between 20-5 ka (with a hiatus during HS1). Together with other paleorecords with low CRI values over central Amazonia (Supplementary Fig.S2, see also Supplementary Table S1 for detailed interpretations), we therefore suggest that central Amazonia underwent slightly drier conditions during HS1 relative to the LGM. A better understanding of this issue depends on new paleoclimate records with high CRI values (e.g., speleothem) from central Amazonia and nearby regions in the future.

Fig.S3. Climatological anomalies between the Heinrich Stadial 1 (HS1) and the Last Glacial Maximum (LGM), as derived from the experiments LGMW-0.2Sv and LGMW in the fully coupled AOGCM respectively (*S11*), including rainfall (shaded, mm/day), 850hPa wind field (vectors, m/s) and sea level pressure (contours, Pa). Good agreement between outputs of the fully coupled AOGCM and the AGCM (Fig.2d in the manuscript), e.g., dry conditions over northernmost South America and wet conditions over the Andes and NE Brazil, suggests that climatological SST variations are a major forcing of South American precipitation changes during HS1. In contrast to both the fully coupled AOGCM (Fig.S3) and the GLB experiment (Fig.2d), our ATL SST experiment shows less precipitation over SE South America (Fig.2c). We suggest that the ATL experiment might underestimate the South Atlantic Convergence Zone rainfall, because it did not include SST changes to the south of 30°S in the Atlantic (see Materials and Methods, supplementary Fig.S4). This map was plotted by using Grid Analysis and Display System (GrADS, Version 2.0.2, http://cola.gmu.edu/grads/grads.php).

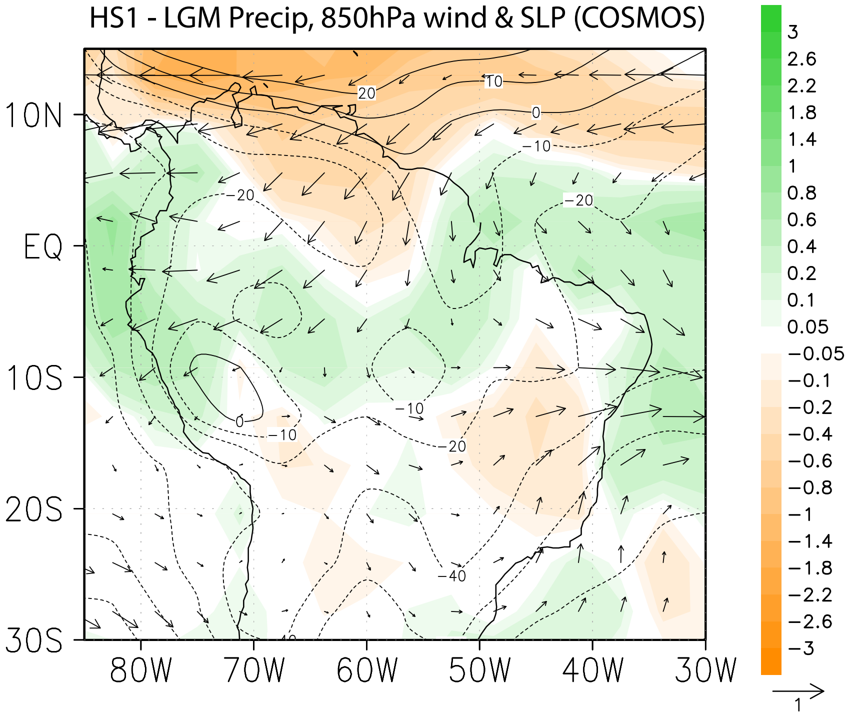

Fig.S4. The COSMOS simulated sea surface temperature (SST) anomalies in the freshwater-hosing experiment (°C) relative to the LGM background experiment (*S11*). The areas outlined within the pink (Atlantic) and black (Pacific) lines were used to perform the ATL SST and EEP SST sensitivity experiments, respectively. Filled circles in the eastern equatorial Pacific denote paleodata-reconstructed SST increases (red) and decreases (blue) during HS1 relative to LGM (data are given in Supplementary Table S2). The map was plotted using the Ocean Data View software (version 4.6.2) (Schlitzer, R., Ocean Data View, http://odv.awi.de, 2015).

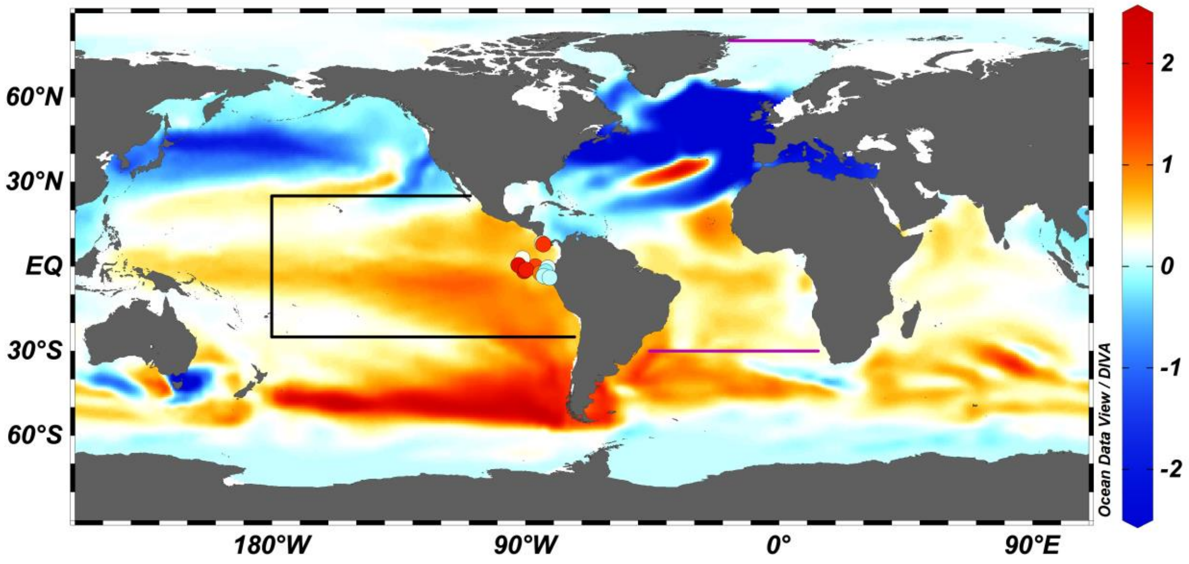

Fig.S5. Schematic map of moisture transport towards the Andes in (a) the ATL experiment and (b) the ATL+EEP experiment. For the ATL experiment, we suppose that less tropical Atlantic moisture is unable to reach the Amazonian Andes directly. In contrast, when the Pacific SST anomalies were forced together with Atlantic SST changes, the tropical Atlantic moisture is further transported from central Amazonia towards the Andes, because of change in sea level pressure gradient between Atlantic and Pacific in the ATL+EEP experiment that leads to an easterly flow from central Amazonia towards the Andes (Fig.2) and then promotes the recycling process of tropical Atlantic moisture. But moisture from both tropical Atlantic and Pacific can hardly cross the Andes due to the steep terrain. The map was plotted by using the ArcGIS software (version 10, https://software.zfn.uni-bremen.de/software/arcgis/).

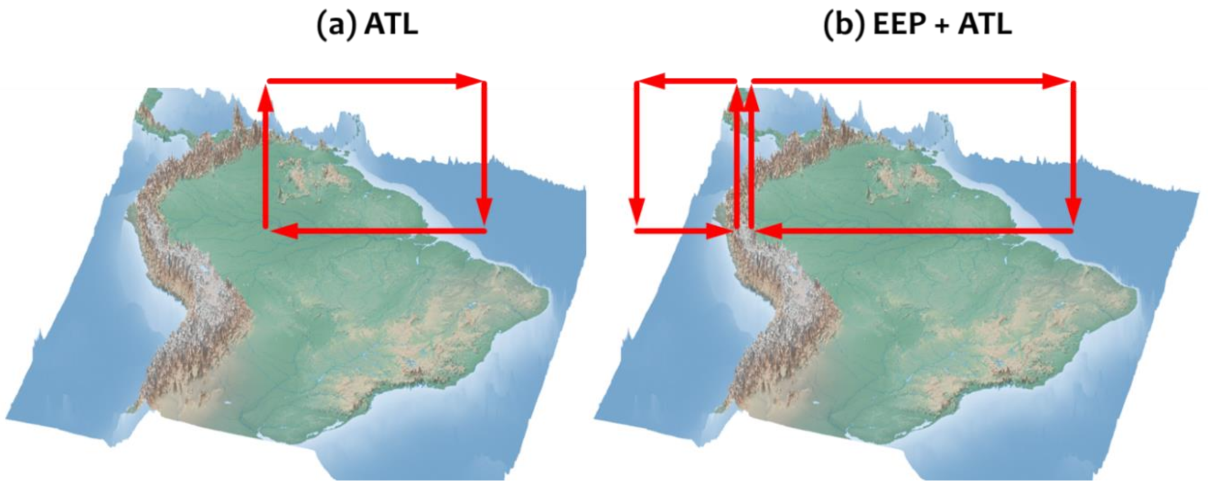

Fig.S6. Wavelet analysis of the El Niño 3.4 SST indices (bounded by 120°W-170°W and 5°S-5°N) from the fully coupled atmosphere-ocean general circulation model (COSMOS, ref. *S11*) for the Last Glacial Maximum (LGM) condition (left) and the freshwater-hosing experiment (right), respectively. The results suggest that the ENSO signal from hosing experiment (thus HS1) is generally characterized by higher frequency (~1 year) and larger variance if compared to the LGM conditions, consistent with other modeling results (ref. *S12-S13*). The wavelet analysis was performed by the Interactive Wavelet Plot (http://ion.exelisvis.com/) and the map was plotted by using the MATLAB software (version R2013b, https://www.marum.de/en/Matlab.html).

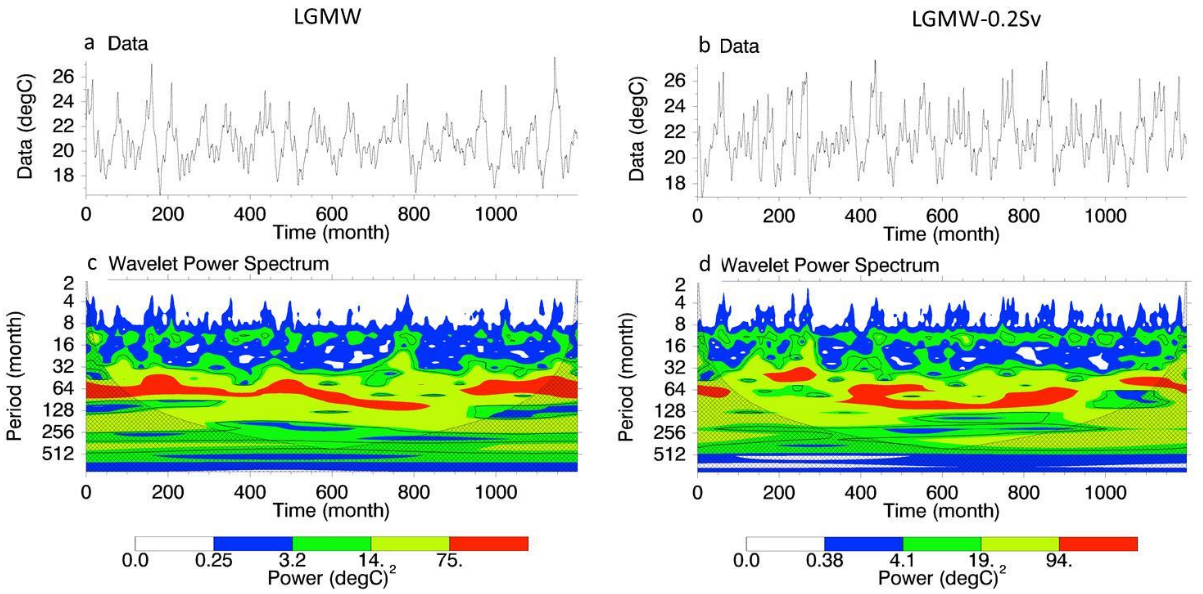

Fig.S7. The simulated seasonality of sea surface temperature (SST, °C) anomalies between the hosing experiment and the Last Glacial Maximum (LGM) control run in COSMOS (*S11*), i.e., December-January-February (DJF, upper left), March-April-May (MAM, upper right), June-July-August (JJA, lower left) and September-October-November (SON, lower right). The map was plotted by the Panoply (version 4.0, http://www.giss.nasa.gov/tools/panoply/).

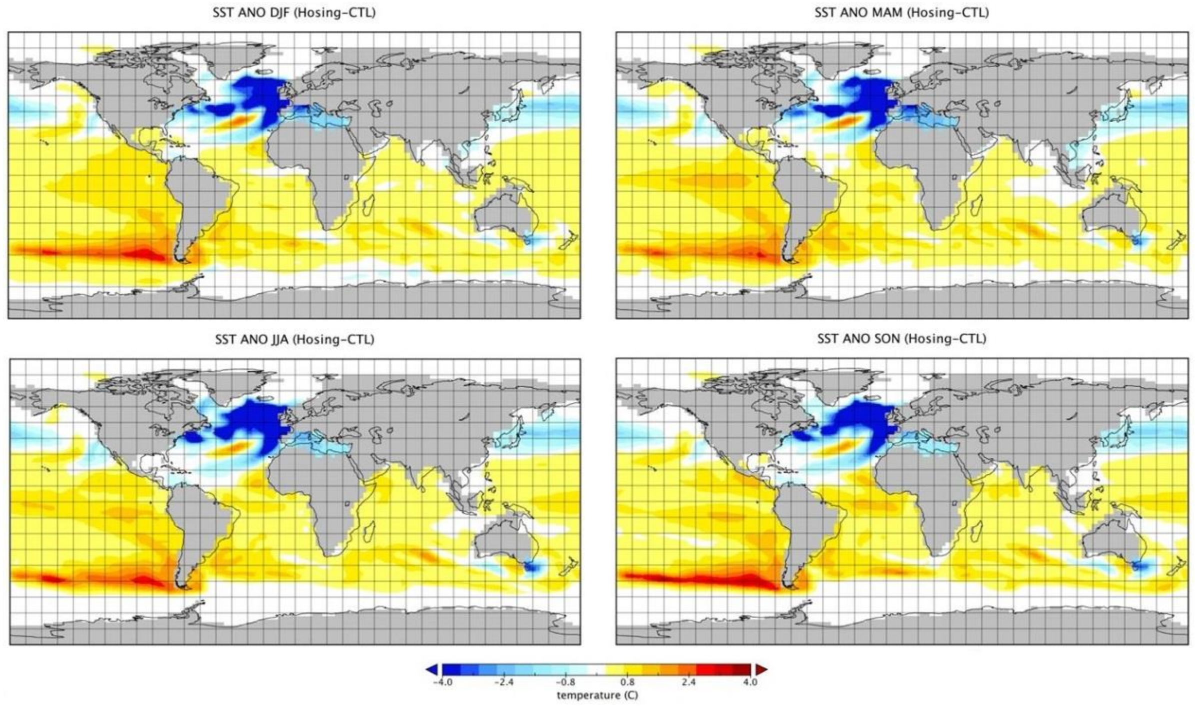

Fig.S8. Results of the atmospheric model sensitivity experiments with equatorial SST anomalies over different regions (other parameters are identical to our original EEP SST experiment), i.e., 180°W-70°W and 15°S-15°N (left), 140°E-70°W and 25°S-25°N (right). Climatology variables include rainfall (shaded, mm/day), 850hPa wind field (vectors, m/s) and sea level pressure (contours, Pa). This map was plotted by using Grid Analysis and Display System (GrADS, Version 2.0.2, http://cola.gmu.edu/grads/grads.php).

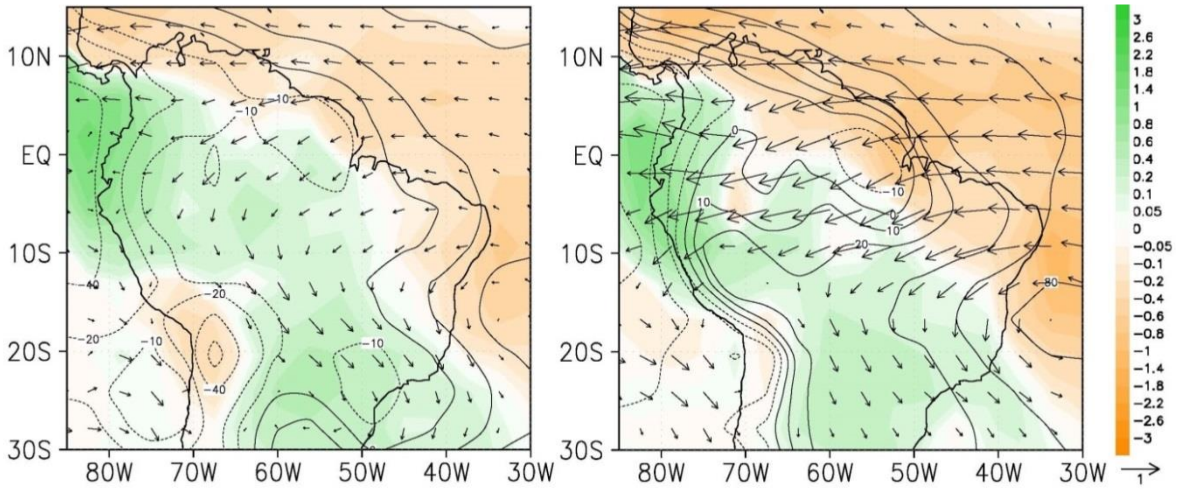

Fig.S9. Orography used in the atmospheric general circulation model (e.g., ECHAM5) to determine the peak of the Andes altitudes, which directly influences the atmospheric circulation pattern via atmospheric gravity wave. Unlike in regional climate models (e.g., Pennsylvania State University/National Center for Atmospheric Research (PSU/NCAR) MM5 (v3.6 model), see ref. *S14*), high resolution topography of the Andes cannot be resolved in this coarse-resolution version of ECHAM5. However, the more accurate altitudes of the Andes may promote the recycling process of the moisture from tropical western Atlantic, but will not lead to overestimation of the contribution of the Pacific Ocean and thus do not change our conclusion. The map was plotted by the Panoply (version 4.0, http://www.giss.nasa.gov/tools/panoply/).

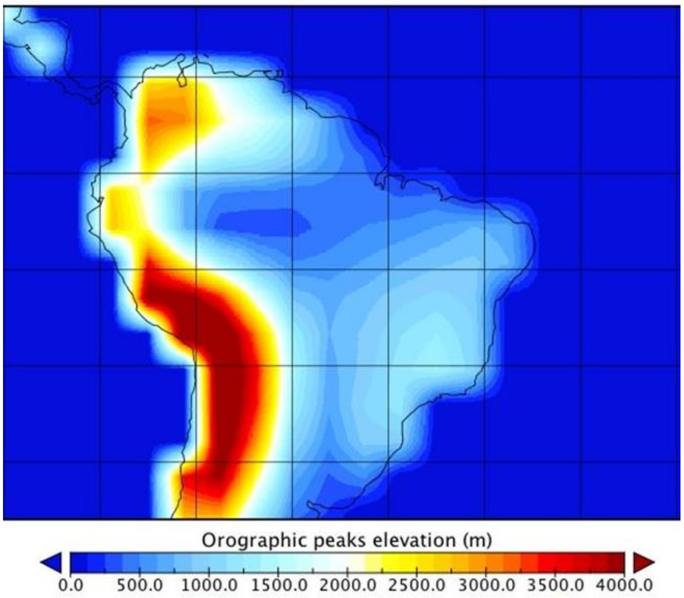

References

S1. Sanchez Goñi, M.F., Harrison, S. P. 2010. Millennial-scale climate variability and vegetation changes during the Last Glacial: Concepts and terminology. Quat. Sci. Rev. 29, 2823-2827.

S2. MARGO Project Members. 2009. Constraints on the magnitude and patterns of ocean cooling at the Last Glacial Maximum. Nature Geosciences 2, 127-132.

S3. Grosjean, M. et al. 2001. A 22,000 14C year BP sediment and pollen record of climate change from Laguna Miscanti (23°S), northern Chile. Global and Planetary Change 28(1-4), 35-51.

S4. Prado, L. F., Wainer, I., Chiessi, C. M., Ledru, M.P., Turcq, B. 2013. A mid-Holocene climate reconstruction for eastern South America. Clim. Past 9, 2117-2133

S5. D'Apolito, C., Absy, M. L., Latrubesse, E. M. 2013. The Hill of Six Lakes revisited new data and re-evaluation of a key Pleistocene Amazon site. Quat. Sci. Rev. 76, 140-155.

S6. Bush, M. B., De-Oliveira, P. E., Colinvaux, P. A., Miller, M. C., Moreno, J. E. 2004. Amazonian paleoecological histories: one hill, three watersheds. Palaeogeogr. Palaeoclimatol. Palaeoecol. 214, 359-393.

S7. Hermanowski, B., Marcondes, L. C., Hermann, B. 2012. Environmental changes in southeastern Amazonia during the last 25,000 yr revealed from a paleoecological record. Quaternary Res. 77, 138-148.

S8. Hermanowski, B., Marcondes, L. C., Hermann, B. 2015. Possible linkages of palaeofires in southeast Amazonia to a changing climate since the Last Glacial Maximum. Veget Hist Archaeobot 24, 279-292.

S9. Carneiro-Filho, A., Schwartz, D., Tatumi, S. H., Rosique, T. 2002. Amazonian Paleodunes Provide Evidence for Drier Climate Phases during the Late Pleistocene–Holocene. Quaternary Res. 58, 205-209 (2002).

S10. Rossetti D. F., Zani, H., Cohen, M. C. L., Cremon, É. H. 2012. A Late Pleistocene–Holocene wetland megafan in the Brazilian Amazonia. Sedimentary Geology 282, 276-293.

S11. Zhang, X., Lohmann, G. , Knorr, G. and Xu, X. 2013. Different ocean states and transient characteristics in the Last Glacial Maximum simulations and implications for deglaciation. Clim. Past 9, 2319-2333.

S12. Merkel, U., Prange, M., Schulz, M. 2010. ENSO variability and teleconnections during glacial climates. Quat. Sci. Rev. 29, 86-100.

S13. Liu, Z. et al. 2014. Evolution and forcing mechanisms of El Niño over the past 21,000 years. Nature 515, 550-553.

S14. Vizy, E. K., Cook, K. H. 2007. Relationship between Amazon and high Andes rainfall. J. Geophys. Res. 112, doi:10.1029/2006JD007980.
